# Supplementary material for: A machine learning-based risk score for prediction of mechanical ventilation in children with dengue shock syndrome: A retrospective cohort study
Source: PLoS One. 2024 Dec 6;19(12):e0315281. doi: 10.1371/journal.pone.0315281 (PMC11623794; doi:10.1371/journal.pone.0315281)
Supplement: S1 Table — (DOCX) [file pone.0315281.s003.docx]

| **Variable names** | **Description** | **Data types** |
| --- | --- | --- |
| Age.year | Patient age by years | Continuous variable |
| Age.year.5 | Patients aged < 5 years | Binary data (1/0) |
| Creatinin.mmol.l | Serum creatinine (µmol/L) in the first 24h of PICU admission | Continuous variable |
| Cumulative.Fluid.Referal.ND2 | Cumulative amount of fluid infused (mL/kg) from referral hospital and 24h of admission in children hospital No.2 | Continuous variable |
| Cumulative.Fluid.Referal.ND2.180 | DSS patients were infused more than 180 mL/kg from referral hospital and 24h of admission in children hospital No.2 | Binary data (1/0) |
| Day.shock | Day of onset of dengue shock since the first manifestation of symptoms | Categorical data |
| DSS.grade | Severity of dengue shock syndrome (DSS) Grade 3- compensated DSS Grade 4- decompensated DSS | Categorical data |
| HCT.nadir | The lowest value of hematocrit (%) during the first 24h of admission | Continuous variable |
| HCT.peak | The highest value of hematocrit (%) during the first 24h of admission | Continuous variable |
| INR | International normalized ratio | Continuous variable |
| Lactate.PICU.admission | Serum lactate (mmol/L) in the first 24h of PICU admission | Continuous variable |
| Obesity | Obesity plotted on WHO growth chart adjusted by patient’s age on admission | Binary data (1/0) |
| Onset.day.shock | Early occurrence of dengue shock ≤ day-4 since the manifestation of the first symptoms | Binary data (1/0) |
| Outcome | Patient’s outcome- severe respiratory failure requiring mechanical ventilation | Binary data (1/0) |
| PLT | Platelet cell count (thousand / mm^3^) | Continuous variable |
| PLT.lower20K | Platelet cell count < 20,000 / mm^3^) | Binary data (1/0) |
| PLT.transfusion | Patients required platelet transfusion | Binary data (1/0) |
| Ratio.Colloid.Crystal | Ratio of colloid to crystalloid fluid infusion | Continuous variable |
| Ratio.Colloid.Crystal.1.6 | Ratio of colloid to crystalloid fluid infusion > 1.6 | Binary data (1/0) |
| Res.rate | Respiratory rate (breaths per minute) | Continuous variable |
| Severe.bleeding | Patient has severe bleeding, defined by WHO Dengue guidelines in 2009 | Binary data (1/0) |
| Severe.hepatitis.WHO | Severe transaminases defined by WHO Dengue guidelines in 2009, as AST or ALT > 1,000 IU/L | Binary data (1/0) |
| Sex | Patient's sex with female (1) & male (0) | Categorical data |
| Sys.shock.index | Systolic shock index (bpm/mmHg) | Continuous variable |
| Underlying.disease | Underlying diseases accompanied at hospital admission | Binary data (1/0) |
| VIS.24h.max | Vasoactive inotropic score (VIS)-highest level during the first 24 hours of PICU admission | Continuous variable |
| VIS24h.over30 | Vasoactive inotropic score (VIS) max levels-during the first 24hours of PICU admission > 30 | Binary data (1/0) |

DSS, Dengue shock syndrome; PICU, pediatric intensive care unit; WHO, World Health Organization
