# Supplementary figures and images for: A machine learning-based risk score for prediction of mechanical ventilation in children with dengue shock syndrome: A retrospective cohort study
Source: PLoS One. 2024 Dec 6;19(12):e0315281. doi: 10.1371/journal.pone.0315281 (PMC11623794; doi:10.1371/journal.pone.0315281)

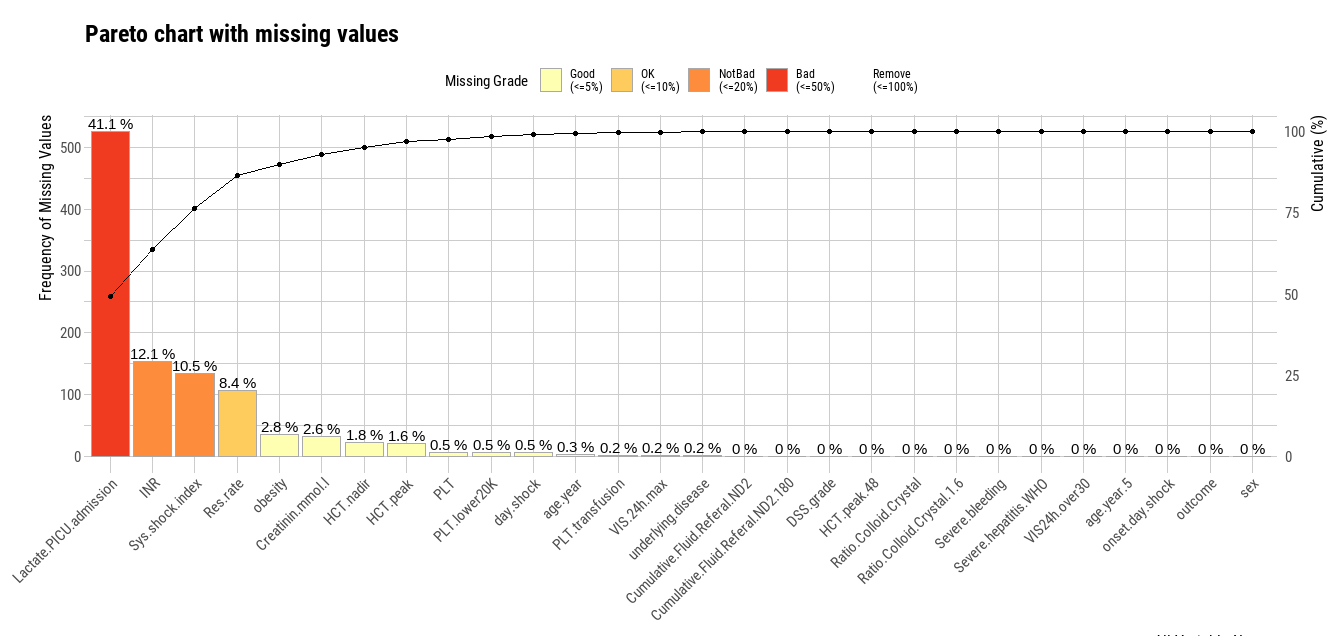

Supplement: S1 Fig — (TIF) [file pone.0315281.s004.tif]
