# Supplementary material for: A machine learning-based risk score for prediction of mechanical ventilation in children with dengue shock syndrome: A retrospective cohort study
Source: PLoS One. 2024 Dec 6;19(12):e0315281. doi: 10.1371/journal.pone.0315281 (PMC11623794; doi:10.1371/journal.pone.0315281)
Supplement: Attachment — Submitted filename: Response to Reviewers.docx [file pone.0315281.s005.docx]

**Response letter to reviewers**

Dear Editors-in-Chief of the PLOS ONE,

We are very thankful for your insightful comments for our submitted manuscript.

**MANUSCRIPT # PONE-D-24-33301**, entitled "A machine learning-based risk score for prediction of mechanical ventilation in children with dengue shock syndrome: A retrospective cohort study"

We are thankful for all peer-review points. These are our responses to all the reviewers’ comments.

**ACADEMIC EDITOR:**

1. Please ensure that extensive language editing is performed prior to submission of the revised manuscript.

**Response:** Yes, we are thankful for your comments. We have checked the language editing in the revision manuscript and resubmitted the latest version as appropriate.

2. Authors use medical terms that are not correct in many places

eg: severe hepatic transaminases - this should be severe transaminitis

**Response:** We are thankful for your comments, and appropriate amendments have been made to the revised manuscript from your suggestions.

3. It is not clear whether this risk score is to be used for patients with respiratory failure or all DSS. clarify.

**Response:** We would like to clarify that this risk score of mechanical ventilation (main study outcome) will be used for all patients with dengue shock syndrome (DSS) who were admitted to the PICU. Among the DSS patients, a certain proportion of children with DSS (170/1278, 13.3%) developed severe respiratory failure that required MV support.

This information has been included in the revision manuscript.

4. Inclusion criteria is not clear.

- Was the model performed on all DSS, all DHF or DHF/DSS with respiratory failure?

- Were they laboratory-confirmed dengue cases?

**Response:** We thank for your comment, and we have made appropriate amendments as follows:

**Lines 128-129** in the revision manuscript.

“*Eligibility criteria were defined as age below 18 years, laboratory-confirmed dengue infection, and the presence of dengue shock syndrome*.”

It is notable the denominator (population) are all PICU-admitted children had laboratory-confirmed dengue infection and DSS, with / without respiratory distress on PICU admission.

5. What were the exclusion criteria? Did you exclude co-infection causing pneumonia and respiratory failure?

**Response:** We are thankful for your comments and have made clarification and amendment as appropriate.

We have made amendments to clarify this point as follows:

“*The exclusion criteria were a lack of serologically confirmed dengue infection and missing data for variables of interest ( ≥ 50%).”*

**Lines 129-130**, in the revision manuscript.

In this study, we aimed to increase the study representativeness and generalizability of the prognostic model. When we developed a baseline prognostic model for mechanical ventilation during 24h of PICU admission, we did not exclude co-infection-causing pneumonia related to mechanical ventilation. Because the ventilator-associated pneumonia (VAP) commonly occurs ≥ 48h -72h after PICU admission (relatively far beyond the baseline timepoint-24h PICU admission)

Additionally, we did not exclude patients with DSS who experienced respiratory failure when they were admitted to the PICU. This is because all referred DSS patients were commonly treated previously within 12h-24h from the province hospital and then transferred to our hospital. Some patients might have respiratory failure and others did not. The most common cause of respiratory failure in the referred DSS patients was due to fluid overload and other dengue-related complications, such as severe bleeding, acute liver failure.

Therefore, with these approaches, we could increase the representativeness and generalizability.

7. Conclusion of the abstract – vague. To manage patients with respiratory failure and predict a poor outcome? Is it actually to predict MV in all severe dengue?

**Response:** We are thankful for your comments. We have made appropriate amendments for clarification, as follows:

*“We developed a robust machine learning-based risk score to estimate the need for MV in hospitalized children with DSS*.”

**Lines 60-61,** in the revision manuscript.

**Reviewer #1:**

I have the following feedback on the write-up. The authors have applied several machine learning models to a real world dataset. Out of many variable, a selected set of variables were chosen for the analysis.
-There are some grammatical errors and typos. Read the manuscript thoroughly and correct them.

**Response:** We are thankful for your comments, and we will make appropriate amendments.

-In the abstract, it says SHAP model explained the significant clinical predictors. However, authors need to mention the machine learning model they used with SHAP model, eg: RF or SVM etc.

**Response:** Thank for your comment. In this study, we utilized several supervised machine

learning models, including Logistic Regression (LR), Random Forest (RF), Support Vector

Machine (SVM), k-Nearest Neighbors (KNN), and Extreme Gradient Boosting (XGBoost), to

predict the need for mechanical ventilation in pediatric patients with dengue shock syndrome. Upon evaluating these models, we found that SVM and RF models demonstrated very good overall performance (AUC, Sensitivity, Specificity, Precision, F1 score, and Accuracy).

We based the optimal performance metrics on determining the SVM model to be additionally analyzed with SHAP.

We add this information in the revised manuscript, **Lines 298-300**

- Authors used variable importance plot with RF model and SVM or RF (not clear) with SHAP to identify the importance features. Authors need to mention why they considered two methods, or why they preferred SHAP method over variable importance given by RF.

**Response:** Currently, there are various supervised models, and it is well noted that under different sampling conditions that one supervised model may be better than other models and vice versa.

- Balancing all performance metrics, RF and SVM showed the two best performance with AUC of 0.96 and high levels of precision and accuracy.
- In addition, the SVM demonstrated slightly better performance than the RF model, in terms of higher sensitivity.

Therefore, we finally determined the SVM as the best performing model. However, the RF model is also considered competitive.

Additionally, SHAP analysis was employed to provide a more detailed and interpretable explanation of how each feature influenced individual predictions. SHAP was preferred because it allows for a consistent and individualized explanation of feature importance across multiple models, offering more granular insights.

We applied the SHAP methodology based on the best supervised model (SVM) for studying the explanation of the fully predefined covariables (also including the 10 most important features identified). In the SHAP model, the 10 features from the SVM model outperformed the remaining risk factors for MV.

Additionally, these are examples to illustrate the point that SHAP values can provide global insights into the feature importance and local explanations of individual predictions of each covariate.

**SHAP for Age**:

As can be seen from this chart, patients with DSS aged < 5 years have an increased risk of MV. The SHAP model can provide us with the distribution of age variable and an explanation for age in the overall prediction of outcome (MV).

Further example for **SHAP model with hematocrit (HCT)** variable in this study:

It can be seen from this SHAP plot that HCT > 48% corresponds to low SHAP values, indicating having a lower risk of MV.

=> Therefore, with all these supported points, the SHAP model was preferred over other supervised models (SVM, RF, KNN,….).

-In line 292, Final predictive model, internal validation and calibration section, it's better to include the name of the final model (RF or SVM etc).

**Response:** We are thankful for your comments.

We used the ten most important predefined covariates in the final SVM model based on its highest performance, which was further analyzed with internal validation and calibration.

We have clarified these points as you suggested, in the revision.

**Lines 273-274**, in the revision manuscript

“*All the predictors from the SVM model demonstrated the utmost significance in the SHAP model*”

**Lines 298-300**, in the revision manuscript

“*The best performance model for MV, with the ten most significant features from the SVM and SHAP models, was internally validated using complete-case analysis and the bootstrap method*.”

-In line 233, authors mentioned that Logistic regression (LR) model has higher rate of false positives. Then they developed a risk score for mechanical ventilation among patients with DSS using LR model in line 304 under "Risk score for mechanical ventilation among patients with DSS" section, which is not justifiable.

- Line 233: “*LR achieved an area under the curve (AUC) of 0.949, with a sensitivity of 0.995, modest specificity of 0.597, precision of 0.941, F1 score of 0.732, and accuracy of 0.942.”*
- Line 304: *“These results confirm that the final predictive model performs well in predicting severe respiratory failure requiring MV among pediatric patients with dengue shock syndrome.”*

**Response:** We thank for your comments. We will answer from point to point.

- We acknowledge that the logistic regression (LR) model has modest specificity (or a higher rate of false positives). This was mainly because of non-linearity of data (skewed data) in this study. Most of the continuous covariables were not normally distributed. We also tried several conventional methods for data normalization, including categorization and log-transformation. This is a well-known limitation of the conventional logistic analysis.
- However, machine learning supervised models can overcome this weakness from conventional analysis when manipulating with non-linearity data. With the similar 10 preselected covariates (used in the LR model above), the supervised machine learning models showed very good performance. In particular, the Support Vector Machine (SVM) is well-known for its capacity to analyze the non-linearity data. Furthermore, we additionally applied the SHAP methodology based on the best supervised model (SVM) for studying the explanation of the fully predefined covariables.
- This is an example illustrating analyzing the peak hematocrit (HCT) with SHAP model:

- It can be seen from this SHAP plot that the HCT is markedly non-linear, clearly observed HCT < 35% and HCT > 54%. Similarly, non-linearity was also significant, regarding covariables of age, INR, and vasoactive inotropic score (VIS).
- Considering the predictive model performance evaluation, we used the criteria developed by Steyerberg EW *et al*. [1,2]. Key criteria for a well-performance model include (1) discrimination (C-statistic or AUC, discrimination slope), (2) Calibration in-the-large, calibration slope, (3) overall performance (Brier score), (4) reclassification and (5) clinical usefulness. Therefore, the specificity parameter is only one contributing indicator for assessing a predictive model, and it is not the main determinant for the evaluation of the model performance [1-5].
- In this study, despite modest specificity of LR model, the final C-statistic of LR model was still high (AUC = 94.9%), as well as precision and accuracy (of approximately 94%). Most importantly, the final predictive model (based on the 10 predefined covariables) for MV showed very good discrimination, calibration of slope, calibration plot, low Brier score as well as the clinical usefulness of the developed model [1,2]. In this study, a high consistency between predicted and observed data was prominently observed (as shown in the **Figure 4)**.

- We constructed a risk score based on logistic regression with complete-case analysis, considering its simplicity and interpretability in clinical practice. Logistic regression provides a straightforward method for computing risk scores that can be easily applied in clinical settings. Notably, we developed the risk score based on references from the Framingham risk score development, and clinical prediction model development and validation methods by Steyerberg EW and Harrell FE Jr [1-11].

**References**

1. Steyerberg EW, Vickers AJ, Cook NR, Gerds T, Gonen M, Obuchowski N, Pencina MJ, Kattan MW. Assessing the performance of prediction models: a framework for traditional and novel measures. Epidemiology. 2010 Jan;21(1):128-38. doi: 10.1097/EDE.0b013e3181c30fb2. PMID: 20010215; PMCID: PMC3575184.
2. Steyerberg EW, Vergouwe Y. Towards better clinical prediction models: seven steps for development and an ABCD for validation. Eur Heart J. 2014 Aug 1;35(29):1925-31. doi: 10.1093/eurheartj/ehu207. Epub 2014 Jun 4. PMID: 24898551; PMCID: PMC4155437.
3. Steyerberg EW, Eijkemans MJ, Harrell FE, Jr, Habbema JD. Prognostic modeling with logistic regression analysis: in search of a sensible strategy in small data sets. *Med Decis Making.* 2001;21:45–56.
4. Steyerberg EW, Harrell FE, Jr, Borsboom GJ, Eijkemans MJ, Vergouwe Y, Habbema JD. Internal validation of predictive models: efficiency of some procedures for logistic regression analysis. *J Clin Epidemiol.* 2001;54:774–781.
5. Steyerberg EW, Borsboom GJ, van Houwelingen HC, Eijkemans MJ, Habbema JD. Validation and updating of predictive logistic regression models: a study on sample size and shrinkage. *Stat Med.* 2004;23:2567–2586.
6. Harrell FE Jr, Lee KL, Califf RM, Pryor DB, Rosati RA. Regression modelling strategies for improved prognostic prediction. Stat Med. 1984 Apr-Jun;3(2):143-52. doi: 10.1002/sim.4780030207. PMID: 6463451.
7. Harrell FE. Regression Modeling Strategies: With Applications to Linear Models, Logistic Regression, and Survival Analysis. New York: Springer; 2001.
8. Harrell FE Jr. Regression Modelling Strategies. Available from: <https://www.r-project.org/nosvn/conferences/useR-2007/tutorials/harrell.html>
9. Steyerberg EW, Eijkemans MJ, Harrell FE, Jr, Habbema JD. Prognostic modelling with logistic regression analysis: a comparison of selection and estimation methods in small data sets. *Stat Med.* 2000;19:1059–1079.
10. Lloyd-Jones DM, Wilson PW, Larson MG, Beiser A, Leip EP, D'Agostino RB, Levy D. Framingham risk score and prediction of lifetime risk for coronary heart disease. Am J Cardiol. 2004 Jul 1;94(1):20-4. doi: 10.1016/j.amjcard.2004.03.023. PMID: 15219502
11. Berry JD, Lloyd-Jones DM, Garside DB, Greenland P. Framingham risk score and prediction of coronary heart disease death in young men. Am Heart J. 2007 Jul;154(1):80-6. doi: 10.1016/j.ahj.2007.03.042. PMID: 17584558; PMCID: PMC2279177.

In this study, there's a significant class imbalance between MV (170) and non-MV groups (1108). Authors can try using class balancing techniques to see if they can further improve the precision and recall along with the accuracies. In addition to that, LASSO models work best for variable selection when there's multicollinearity and the usual practice is to check for multicollinearity before applying LASSO models. Out of many variable, a selected set of variables were chosen for the analysis using some ad-hoc method and it is recommended to use a proper variable selection method (from all non-missing variables).

**Response:**

The proportion of MV among the DSS population was 170 / 1278 (13.3%), which is considered to have relatively sufficient statistical power for predictive model development. Importantly, this figure (MV prevalence of 13.3% among DSS) truly reflects the real-life data of patients with dengue shock syndrome, that is consistent with the previously published data [1]. Therefore, we do not think that the MV imbalance is considerable in this study. We conservatively believe that it is not necessary to perform oversampling methods (SMOTE...etc).

Also, we did not find any multicollinearity when analyzing the LASSO. Additionally, all covariates were independent. We recognized that LASSO seemed to be too stringent in variable selection, and eliminated some significant variables that are well known in disease pathogenesis and clinical importance. As stated in the manuscript, we combined and balanced different methods of LASSO, clinical knowledge, and the medical literature for preselecting the covariates.

**Reference**

[1] Nguyen TT, Nguyen DT, Vo TT, et al. Associations of obesity and dengue-associated mortality, acute liver failure and mechanical ventilation in children with dengue shock syndrome. Medicine (Baltimore). 2023 Nov 17;102(46):e36054. doi: 10.1097/MD.0000000000036054. PMID: 37986332; PMCID: PMC10659721.

**Reviewer #2:**

This is an interesting analysis that attempts to incorporate artificial intelligence into clinical practice. However, the flow of information presented in the text can be improved.
I would like to suggest the following.

**Introduction section**
1. The authors should verify the objective of this study. In lines 106 and 127, different aims have been mentioned.

**Line 106**: “*We aimed to employ machine learning to develop robust prognostic models and a risk scoring system to predict the risk of mechanical ventilation in hospitalized children with dengue shock syndrome”*

**Line 127**: “*The primary aim of this study was to identify the determinants of severe respiratory failure requiring mechanical ventilation in PICU-admitted children with dengue shock syndrome”*

**Response:** We thank for your comment. We will make clarifications and amendments.

We aimed to develop a machine-learning risk score to predict MV among DSS patients. To achieve this, there are two main steps. In the first step, we identified the most significant determinants/predictors associated with severe respiratory failure requiring mechanical ventilation support. In the second step, from the identified predictors we constructed a machine learning-based risk score to estimate the risk of MV among PICU-admitted children with DSS.

=> We have made appropriate amendments to address these two ideas in the revised manuscript.

“*This study aimed to identify the most significant predictors of MV and develop a robust machine learning-based risk-scoring system to predict the need for MV in hospitalized children with DSS.”*

**Lines 106-108 & Lines 126-128** in the revision manuscript.

**Methods section**
2. Contradictory information on the study population. Please verify ( refer to line 129).

Line 129: “*This study included pediatric patients admitted to the PICU with severe respiratory failure necessitating mechanical ventilation due to severe dengue infection*”

**Response:**

2. We are thankful for your comment. We make a correction from your suggested point:

**=>** This information will be corrected with the appropriate inclusion and exclusion criteria in the revised manuscript, as below:

“*The eligibility criteria were age < 18 years, laboratory-confirmed dengue infection, and the presence of dengue shock syndrome [2]. The exclusion criteria were a lack of serologically confirmed dengue infection and missing data for variables of interest (≥ 50%)*.”

**Lines 128-130,** in the revision manuscript.

3. It is unclear how participants were recruited from the database and who was eligible for this particular study. Please explain.

**Response:**

This study stemmed from an original published study [4], including approximately 2,000 patients with DSS admitted to the PICU. We identified 1,278 patients with complete data of interest, included in the analysis from machine learning models. The remaining patients (approx. 720) were excluded owing to significant missing data of predefined covariates of interest ( ≥ 50%) and/or lack of serologically confirmed dengue infection.

We have added appropriate inclusion and exclusion criteria in the revised manuscript.

**Lines 128-130, & Lines 211-212** in the revision manuscript.

**Reference:**

[4] Nguyen Tat T, Vo Hoang-Thien N, Nguyen Tat D, Nguyen PH, Ho LT, Doan DH, et al. Prognostic values of serum lactate-to-bicarbonate ratio and lactate for predicting 28-day in-hospital mortality in children with dengue shock syndrome. Medicine (Baltimore). 2024; 103 (17): e38000. doi: 10.1097/MD.0000000000038000. PMID: 38669370; PMCID: PMC11049702

4. Out of 82 variables in eligible participants, 28 variables were initially selected for analysis. Describe the rationale for selecting only some variables for analysis (lines 139 & 140).

Lines 139-140: *From the original dataset, we identified 1,278 eligible participants with 82 variables [4]. We extracted 28 variables of interest that had a missing rate of < 50% and were consistent with disease pathogenesis* (**S1 Table**).

**Response:**

The original dataset stemmed from the original published study [4], which encompassed 82 variables including ID, demographic information, clinical and laboratory data. Unnecessary data were excluded, such as ID...etc. Subsequently, 28 relevant variables of clinical and laboratory data were retained, as shown in the **S1 table**. These 28 relevant variables were carefully predefined and preselected, based on our clinical knowledge, pathogenesis and medical literature review [3-5, 8-11].

**References:**

[3] Nguyen TT, Nguyen DT, Vo TT, Dang OT, Nguyen BT, Pham DT, et al. Associations of obesity and dengue-associated mortality, acute liver failure and mechanical ventilation in children with dengue shock syndrome. Medicine (Baltimore). 2023; 102 (46): e36054. doi: 10.1097/MD.0000000000036054. PMID: 37986332; PMCID: PMC10659721

[4] Nguyen Tat T, Vo Hoang-Thien N, Nguyen Tat D, Nguyen PH, Ho LT, Doan DH, et al. Prognostic values of serum lactate-to-bicarbonate ratio and lactate for predicting 28-day in-hospital mortality in children with dengue shock syndrome. Medicine (Baltimore). 2024; 103 (17): e38000. doi: 10.1097/MD.0000000000038000. PMID: 38669370; PMCID: PMC11049702

[5] Sachdev A, Pathak D, Gupta N, Simalti A, Gupta D, Gupta S, et al. Early Predictors of Mortality in Children with Severe Dengue Fever: A Prospective Study. Pediatr Infect Dis J. 2021; 40 (9): 797-801. doi: 10.1097/INF.0000000000003179. PMID: 34321449

[8] Preeprem N, Phumeetham S. Paediatric dengue shock syndrome and acute respiratory failure: a single-centre retrospective study. BMJ Paediatr Open. 2022 ; 6(1): e001578. doi: 10.1136/bmjpo-2022-001578. PMID: 36645744; PMCID: PMC9668030

[9] Vo LT, Nguyen DT, Tran TN, Tran HH, Đoan TT, Pham TN, et al. Pediatric Profound Dengue Shock Syndrome and Use of Point-of-Care Ultrasound During Mechanical Ventilation to Guide Treatment: Single-Center Retrospective Study, 2013-2021. Pediatr Crit Care Med. 2024; 25 (4): e177-e185. doi: 10.1097/PCC.0000000000003413. Epub 2023 Nov 15. PMID: 37966344; PMCID: PMC10986784

[10] Chen CM, Chan KS, Yu WL, Cheng KC, Chao HC, Yeh CY, et al. The outcomes of patients with severe dengue admitted to intensive care units. Medicine (Baltimore). 2016; 95(31): e4376. doi: 10.1097/MD.0000000000004376. PMID: 27495047; PMCID: PMC4979801

[11] Nguyen TT, Le NT, Nguyen NM, Do VC, Trinh TH, Vo LT. Clinical features and management of children with dengue-associated obstructive shock syndrome: A case report. Medicine (Baltimore). 2022; 101(43): e31322. doi: 10.1097/MD.0000000000031322. PMID: 36316901; PMCID: PMC9622697

5. Was there a uniformity of criteria used for starting mechanical ventilation at the PICU?
The authors should clarify this. If different clinicians used different criteria, there is a potential for bias in this analysis.

**Response:** We are thankful for your important comment.

We used the same criteria for starting mechanical ventilation in patients with dengue shock and severe dengue according to the national guidelines. All PICU doctors used the same guidelines on the indications of mechanical ventilation for DSS patients. Therefore, there is a lower likelihood of bias, as you mentioned.

We used our national dengue guidelines from the Vietnamese Ministry of Health [1] for indications of mechanical ventilation for the entire cohort, as follows:

*Indications for mechanical ventilation in severe dengue patients* [1]:

- Dengue shock syndrome (DSS) with severe respiratory failure:
- Failed with NCPAP (nasal continuous positive airway pressure) and paracentesis due to from abdominal compartment syndrome (ACS)
- Pulmonary edema / fluid overload, failed with NCPAP, vasopressors
- ARDS, failed with NCPAP
- Failed with NCPAP, substantial pleural and abdominal cavity effusions on day 3,4,5 after disease onset.
- Severe and progressive acute liver failure, on NCPAP
- Ongoing dengue shock, experiencing tachypnea, intercostal retraction with NCPAP, despite SpO2 ≥ 95%, (fluid infused ≥ 7ml/kg/hour in many hours)

We additionally added this information in the supplementary data **(S1 File).**

**Reference**

[1] Vietnamese Ministry of Health 2011 dengue guidelines. Available from: <https://thuvienphapluat.vn/van-ban/The-thao-Y-te/Quyet-dinh-458-QD-BYT-huong-dan-chan-doan-dieu-tri-sot-xuat-huyet-Dengue-120583.aspx>

6. Data on participant numbers should also be included in the methods section (refer lines 203 & 204).

Lines 203 & 204: *The study included 1278 children with DSS admitted to the PICU, of which 170 patients (13.3%) experienced severe respiratory failure, requiring MV and the remaining 1108 did not*

**Response:**

Between 2013 and 2022, approximately 2,000 children with DSS were admitted to the PICU, and of whom 1,278 patients met the eligibility criteria and were included in the analysis.

From your suggestion, we have added the number of participants to the revised manuscript. **Lines 211-212,** in the revision manuscript**.**

**Results section**
1. The authors have given general statements in the results section. For example “ patients who required MV were generally younger and more females than males…”. Please describe in terms of mean or median age and sex percentage (refer line 206).

**Response:** We thank for your suggestion. We have presented the suggested data in Table 1. We think it is not necessary to describe them in detail because the readers can easily read Table 1 for this information.

2. Please define the terms used in the text – critical bleeding, severe transaminitis, etc.

**Response:** We are thankful for your suggested points. Critical (severe) bleeding and severe transaminitis were defined according to the 2009 WHO Dengue guidelines. We have added these points to the revised manuscript as follows.

**Study definitions**

*“According to the World Health Organization (WHO) dengue guidelines (2009), laboratory-confirmed dengue infection was defined by the presence of dengue-IgM antibodies or a positive nonstructural 1 antigen test [2]. Dengue shock syndrome and severe bleeding were diagnosed according to the WHO dengue 2009 guidelines [2]. Severe transaminitis was defined as elevated aspartate aminotransferase (AST) and/or alanine aminotransferase (ALT) levels of ≥ 1,000 IU/L [2]*.”

**Lines 134-139,** in the revision manuscript.

**Reference**

**[2]** WHO. Dengue: Guidelines for Diagnosis, Treatment, Prevention and Control. Geneva: World Health Organization, 2009. Available from: <https://iris.who.int/handle/10665/44188>

3. How did the authors grade the severity of dengue infection?

**Response:** We defined the grade of the severity of dengue infection, in accordance with the WHO Dengue guidelines in 2009 [2].

We have clarified this point that we would like to state the grade of dengue shock syndrome (DSS) severity (instead of the severity of dengue), according to the WHO guideline (2009):

- Compensated DSS
- Decompensated DSS

We have corrected this information in **Table 1 (row 7**) in the revision manuscript.

**Reference**

[2] WHO. Dengue: Guidelines for Diagnosis, Treatment, Prevention and Control. Geneva: World Health Organization, 2009. Available from: <https://iris.who.int/handle/10665/44188>

4. **Table 1**
• The naming of the 2nd and 3rd columns is confusing. It sounds as if one group received a form of non-mechanical ventilation. Therefore, name them appropriately. For example, MV required and MV not required.
• Mention the statistical tests used to derive p value in table 1.

**Response:** We thank for your suggested points.

From the clinician’s viewpoint, we still believe that mechanical ventilation (MV) and non-mechanical ventilation (non-MV) are appropriate and easy to understand. We are determined to retain the data presented.

In addition, we have added the statistical tests in the footnote of **Table 1** in the revised manuscript.

“*Summary statistics are presented as median (interquartile range, IQR) for continuous variables and frequency (%) for categorical variables.* *Two-sided, two-sample* t *tests were used to compare means of continuous variables. The Chi-square test and adjusted Fisher exact test were used to compare categorical variables*.”

5. Please describe how sub-coefficients were used to derive a risk score (refer line 307).

Line 307: “ The risk score table was developed based on the subcoefficients of of all ten significant predictors.”

**Response:** We are thankful for your consideration. We will make a clarification for this point.

We mainly based the risk score development method, originating from Framingham Risk Score development [1,2], on constructing the risk score for dengue MV among DSS patients.

**References**

1. Lloyd-Jones DM, Wilson PW, Larson MG, Beiser A, Leip EP, D'Agostino RB, Levy D. Framingham risk score and prediction of lifetime risk for coronary heart disease. Am J Cardiol. 2004 Jul 1;94(1):20-4. doi: 10.1016/j.amjcard.2004.03.023. PMID: 15219502
2. Berry JD, Lloyd-Jones DM, Garside DB, Greenland P. Framingham risk score and prediction of coronary heart disease death in young men. Am Heart J. 2007 Jul;154(1):80-6. doi: 10.1016/j.ahj.2007.03.042. PMID: 17584558; PMCID: PMC2279177.

We answer your query of “how sub-coefficients were used to derive a risk score” in details.

**In general, for the outcome Y (MV), the logit equation can be described as:**

(Source: <https://towardsdatascience.com/how-are-logistic-regression-ordinary-least-squares-regression-related-1deab32d79f5>)

In this study, from the adjusted multivariable logistic analysis, we have the results for subcoefficients and intercepts from analysis with R statistical software as below.

**R output:**

```
summary(multimodel.final)

Call:
glm(formula = outcome ~ age.year.5 + sex + Cumulative.Fluid.Referal.ND2.180 +
    Ratio.Colloid.Crystal.1.6 + PLT.lower20K + Severe.bleeding +
    Severe.hepatitis.WHO + onset.day.shock + log2.VIS.24h.max +
    HCT.peak.48, family = binomial, data = Mechanical.Ventilation)

Coefficients:
                                     Estimate Std. Error z value Pr(>|z|)
(Intercept)                           -5.6172     0.4559 -12.321  < 2e-16 ***
age.year.5TRUE                         1.1425     0.3207   3.563 0.000367 ***
sex1                                   0.8074     0.2902   2.783 0.005394 **
Cumulative.Fluid.Referal.ND2.180TRUE   2.6179     0.3201   8.179 2.87e-16 ***
Ratio.Colloid.Crystal.1.6TRUE          0.8727     0.2967   2.941 0.003268 **
PLT.lower20K1                          0.2642     0.3296   0.801 0.422847
Severe.bleeding1                       3.1710     0.4144   7.651 1.99e-14 ***
Severe.hepatitis.WHO1                  1.4010     0.3854   3.635 0.000278 ***
onset.day.shock1                       0.5101     0.2898   1.760 0.078406 .
log2.VIS.24h.max                       1.1380     0.1475   7.717 1.19e-14 ***
HCT.peak.48TRUE                       -0.4467     0.2872  -1.555 0.119873
---
Signif. codes:  0 ‘***’ 0.001 ‘**’ 0.01 ‘*’ 0.05 ‘.’ 0.1 ‘ ’ 1

(Dispersion parameter for binomial family taken to be 1)

    Null deviance: 995.86  on 1268  degrees of freedom
Residual deviance: 373.81  on 1258  degrees of freedom
  (9 observations deleted due to missingness)
```

**Hence, we have:**

- Intercept = -5.6172 and Beta (Subcoefficients) of all covariables in the risk score are withdrawn from the multivariable logistic regression.

Next, we have presented the analysis output in the **Table 3** (in the submitted manuscript).

**Table 3**. Risk score to predict mechanical ventilation among children with dengue shock syndrome during the first 24h of PICU admission

| **Predictors** | **Subcategory** | **Reference values** | **Sub-coefficients** | **SE** | **Risk score** |
| --- | --- | --- | --- | --- | --- |
| Age (years) | ≥ 5 | Reference | 0 | - | 0 |
|  | < 5 | - | 1.143 | 0.321 | 4 |
| Female patients | No | Reference | 0 | - | 0 |
|  | Yes | - | 0.807 | 0.290 | 3 |
| Cumulative amount of fluid infused, (mL/kg) | ≤ 180 | Reference | 0 | - | 0 |
|  | > 180 | - | 2.618 | 0.320 | 10 |
| Ratio of colloid-to-crystalloid fluid infusion | < 1.6 | Reference | 0 | - | 0 |
|  | ≥ 1.6 | - | 0.873 | 0.297 | 3 |
| **Platelet counts**  **(< 20 x 10^9^/L)** | **No** | **Reference** | **0** | **-** | **0** |
|  | **Yes** | **-** | **0.264** | 0.329 | **1** |
| Severe bleeding | No | Reference | 0 | - | 0 |
|  | Yes | - | 3.171 | 0.414 | 12 |
| + 01-log2-increase in VIS during first 24h of admission | No | Reference | 0 | - | 0 |
|  | Yes | - | 1.138 | 0.148 | 4 |
| Severe transaminitis | No | Reference | 0 | - | 0 |
|  | Yes | - | 1.789 | 0.385 | 7 |
| Onset day of shock | > day 4 | Reference | 0 | - | 0 |
|  | ≤ day 4 | - | 0.510 | 0.290 | 2 |
| Peak hematocrit (%) | ≤ 48% | Reference | 0 | - | 0 |
|  | > 48% | - | - 0.447 | 0.287 | -2 |
| *Range of risk score* | From -2 to 46 | | | | |

**Notes:** Sub-coefficients stemmed from the slopes of multivariable logistic model (complete-case analysis); PICU, pediatric intensive care unit; SE, standard errors of coefficients; VIS, vasoactive inotropic score

**The logit equation for outcome (MV) can be shown as:**

**(1)**

Logit (Y) = intercept (-5.6172)+ Beta (Platelet < 20 x 10^9^/L)*Platelet (< 20 x 10^9^/L, Yes =1 vs No = 0, reference) + Beta (patient’s age)*Age (<5 years vs ≥ 5 years)+.......+ Beta (Peak hematocrit)* HCT (HCT ≤ 48% = 0, vs HCT > 48% =1)

*This can be written as:*

Logit (Y) = intercept (-5.6172)+ **0.264***Platelet (< 20 x 10^9^/L, Yes =1 vs No = 0, reference) + 1.143*Age (<5 years vs ≥ 5 years)+...+ (- 0.447)* HCT level (HCT ≤ 48% = 0, vs HCT > 48% =1)

=> We selected *low platelet counts* (< 20 x 10^9^/L) subcoefficient because it represented the *lowest* *absolute value*.

We then divided all the remaining covariate subcoefficients to platelet subcoeffecient (0.264).

*Therefore, the logit equation (1) can be equally to this new converted equation:*

**(2)**

Logit (Y) = 0.264 * [ (intercept (-5.6172) /0.264) + **1** *(Platelet < 20K cells/mm3)*Platelet <20 cells/mm3 (Yes =1/No = 0) + **(1.143 / 0.264)** *Age (<5 years vs ≥ 5 years) +…+ (**-0.447 / 0.264**)* HCT level (HCT ≤ 48% = 0, vs HCT > 48% =1)

- *The above equation (2) can also be rewritten as:*

Logit (Y) = 0.264 * [(intercept (-5.6172) / 0.264) (newly adjusted intercept) + **1***(Platelet < 20 cells/mm3) + **4*** Age +….+ (-**2**)* HCT)

=> The bold numbers above are the risk score for covariates.

*Abbreviate the above equation, we have:*

Logit (Y) = newly adjusted intercept + [Total **RISK SCORE** (of 10 final covariates identified from SHAP & SVM models) ]

=> Then we calculate the probability of outcome MV (with *PLOGIS* function on R command)

Notably, we rounded up the risk score, eg, 2.87 was rounded up to 3 points. Theoretically, there might be some loss from this rounding; however, this could be tested in the calibration of slopes and calibration plot, showing no influence on overall effect.

6. Explain how to use risk score practically.

**Response:** We are thankful for your query, and we will clarify how to use the risk score practically.

We will apply the developed risk score for DSS children admitted to the PICU, to determine if patients can have a high or low risk of requiring mechanical ventilation. This also indicates the severity of respiratory failure, reported as the strong predictor of death in DSS patients.

For example, upon PICU admission, if a female DSS patient had age = 8 years (> 5 years), infused intravenous fluid 200 mL/kg from the referral hospital, with the ratio of colloid to crystalloid 1.8, platelet count 15 x 10^9^ / L, no sign of severe bleeding, without vasopressor support (VIS = 0), AST = 100 IU/L, ALT = 120 IU/L, at day 5 since disease onset, hematocrit = 45% on admission.

- From the **Table 3**, we can calculate the total risk score of MV for this patient is 17. Next, we check the risk score chart (in the **Fig 5**), to see the corresponding probability for the total risk score of 17.

Therefore, in this patient with the total risk score of 17 (x-axis), and the probability of the need for MV support is 25% (y-axis).

This risk score can be integrated into an app on smartphones for clinicians to use at the bedside to estimate the risk of mechanical ventilation in DSS patients upon PICU admission.

7. Table 3
• Define severe bleeding and severe transaminitis in the main text as well. ( There are defined in supporting documents)
• It is necessary to decide on a cut-off value for severe transaminitis to derive the risk score.

**Response:** We are thankful for your suggested points. Critical (severe) bleeding and severe transaminitis were defined according to the WHO Dengue guidelines in 2009 [2]. We have added these points in the revision manuscript, as below:

**Study definitions**

*“According to the World Health Organization (WHO) dengue guidelines (2009), laboratory-confirmed dengue infection was defined by the presence of dengue-IgM antibodies or a positive nonstructural 1 antigen test [2]. Dengue shock syndrome and severe bleeding were diagnosed according to the WHO dengue 2009 guidelines [2]. Severe transaminitis was defined as elevated aspartate aminotransferase (AST) and/or alanine aminotransferase (ALT) levels of ≥ 1,000 IU/L [2]*.”

**Lines 134-139,** in the revision manuscript.

=> We also performed statistical analysis to determine the various cut-off values for AST/ALT. Finally, we determined that AST/ALT ≥ 1,000 UI/L (defined as severe transaminitis according to the WHO Dengue 2009 guidelines) was the optimal cut-off point in this study.

**Reference**

[2] WHO. Dengue: Guidelines for Diagnosis, Treatment, Prevention and Control. Geneva: World Health Organization, 2009.

**Discussion section**
1. In Table 3, it is mentioned thrombocytopenia is a risk factor but, in the text, it is said thrombocytopenia needing platelet transfusions is a risk factor. Verify if thrombocytopenia per se or thrombocytopenia requiring platelet transfusions was a risk factor for MV.

**Response:** We are thankful for comment. We believe the language expression is a barrier to expressing our complete statement. We clarify this point and make amendment as appropriate.

Platelet transfusion has been reported as a strong predictor of severe dengue from various published studies. In clinical practice, we only transfused platelets in patients who were experiencing severe bleeding with thrombocytopenia, or who required invasive procedures such as intubation, central line placement, or abdominal decompression. Platelet transfusion indicates ongoing severe bleeding status of severe dengue patients. Notably, severe bleeding is a major predictor of mortality in dengue-infected patients [1-3]. Additionally, low platelet counts (particularly, platelet counts < 20 x 10^9^/L) have also been well reported as a significant predictor of dengue severity [4-6].

*Considering the predictive model of MV among DSS patients in our study:*

We balanced the parsimonious principle and generalizability in developing a prognostic model. We determined to use the thrombocytopenia (platelet counts < 20 x 10^9^/L) in our final predictive model. This variable (platelet counts < 20 x 10^9^/L) in predicting the need for mechanical ventilation, has been well supported from our RF & SHAP analyses, shown in the **Figures 2 and 3** in the following.

**Figure 2**. Features of importance from Random Forest (RF) model

**Figure 3**. SHAP model

=> Both Random Forest and SHAP models clearly showed that thrombocytopenia (platelet counts < 20 x 10^9^/L) is a crucial predictor of mechanical ventilation among DSS patients.

To summarize, thrombocytopenia (platelet cell counts < 20 x 10^9^/L) is a significant predictor of MV among DSS patients. Moreover, platelet transfusion is a crucial predictor of severe dengue. Considering the generalizability, we finally selected thrombocytopenia (platelet cell counts < 20 x 10^9^/L) in our final predictive model.

**Reference**

1. Kaur G, Kumar V, Puri S, Tyagi R, Singh A, Kaur H. Predictors of dengue-related mortality in young adults in a tertiary care centre in North India. J Family Med Prim Care. 2020 Feb 28;9(2):694-697. doi: 10.4103/jfmpc.jfmpc_605_19. PMID: 32318404; PMCID: PMC7114013.
2. John KJ, Gunasekaran K, Prasad JD, Mathew D, Das S, Sultan N, Abraham AM, Iyyadurai R. Predictors of Major Bleeding and Mortality in Dengue Infection: A Retrospective Observational Study in a Tertiary Care Centre in South India. Interdiscip Perspect Infect Dis. 2019 Sep 4;2019:4823791. doi: 10.1155/2019/4823791. PMID: 31565054; PMCID: PMC6746148.
3. Md-Sani SS, Md-Noor J, Han WH, Gan SP, Rani NS, Tan HL, Rathakrishnan K, A-Shariffuddin MA, Abd-Rahman M. Prediction of mortality in severe dengue cases. BMC Infect Dis. 2018 May 21;18(1):232. doi: 10.1186/s12879-018-3141-6. PMID: 29783955; PMCID: PMC5963083.
4. Sangkaew S, Ming D, Boonyasiri A, Honeyford K, Kalayanarooj S, Yacoub S, Dorigatti I, Holmes A. Risk predictors of progression to severe disease during the febrile phase of dengue: a systematic review and meta-analysis. Lancet Infect Dis. 2021 Jul;21(7):1014-1026. doi: 10.1016/S1473-3099(20)30601-0. Epub 2021 Feb 25. PMID: 33640077; PMCID: PMC8240557.
5. Nguyen Tat T, Vo Hoang-Thien N, Nguyen Tat D, Nguyen PH, Ho LT, Doan DH, et al. Prognostic values of serum lactate-to-bicarbonate ratio and lactate for predicting 28-day in-hospital mortality in children with dengue shock syndrome. Medicine (Baltimore). 2024 Apr 26;103(17):e38000. doi: 10.1097/MD.0000000000038000. PMID: 38669370; PMCID: PMC11049702.
6. Tsheten T, Clements ACA, Gray DJ, Adhikary RK, Furuya-Kanamori L, Wangdi K. Clinical predictors of severe dengue: a systematic review and meta-analysis. Infect Dis Poverty. 2021 Oct 9;10(1):123. doi: 10.1186/s40249-021-00908-2. PMID: 34627388; PMCID: PMC8501593.

2. Have the platelet transfusions caused respiratory failure by fluid overload?

**Response:** We thank for this comment.

We transfused highly concentrated platelets, equivalent to a negligible volume, which could not cause fluid overload and respiratory failure.

Regarding this study analysis, we also combined the volume of platelet transfusion in the predefined covariate “**cumulative volume of fluid infusion**”, analyzed in the machine learning models.

3. Investigating if blood transfusions were a risk factor for MV may be interesting.

**Response:** We are thankful for your suggested points.

Notably, severe bleeding has been reported to be associated with poor outcomes in DSS patients [1-6], and it is also a diagnostic criterion for severe DSS according to the WHO 2009 classification [7]. According to WHO 2009 guidelines, blood transfusion should be administered only in cases with clinically suspected severe bleeding. Thus, choosing the variable of critical bleeding (instead of blood transfusion) is consistent with general recommendations based on the WHO dengue guidelines. Additionally, critical bleeding is well evidenced, widely accepted, and used in the medical literature [1-7].

In this study analysis, we did combine the volume of blood transfusion in the predefined covariate of “**cumulative volume of fluid infusion**”, analyzed in the machine learning models.

We also think your suggested idea for investigating “*blood transfusions were a risk factor for MV may be interesting*” which is more appropriate in another study, because it is beyond the scope of our study. We will consider your suggestion for a future prospective study.

**Reference**

1. John KJ, Gunasekaran K, Prasad JD, Mathew D, Das S, Sultan N, Abraham AM, Iyyadurai R. Predictors of Major Bleeding and Mortality in Dengue Infection: A Retrospective Observational Study in a Tertiary Care Centre in South India. Interdiscip Perspect Infect Dis. 2019 Sep 4;2019:4823791. doi: 10.1155/2019/4823791. PMID: 31565054; PMCID: PMC6746148.
2. Kaur G, Kumar V, Puri S, Tyagi R, Singh A, Kaur H. Predictors of dengue-related mortality in young adults in a tertiary care centre in North India. J Family Med Prim Care. 2020 Feb 28;9(2):694-697. doi: 10.4103/jfmpc.jfmpc_605_19. PMID: 32318404; PMCID: PMC7114013.
3. Md-Sani SS, Md-Noor J, Han WH, Gan SP, Rani NS, Tan HL, Rathakrishnan K, A-Shariffuddin MA, Abd-Rahman M. Prediction of mortality in severe dengue cases. BMC Infect Dis. 2018 May 21;18(1):232. doi: 10.1186/s12879-018-3141-6. PMID: 29783955; PMCID: PMC5963083.
4. Medagama A, Dalugama C, Meiyalakan G, Lakmali D. Risk Factors Associated with Fatal Dengue Hemorrhagic Fever in Adults: A Case Control Study. Can J Infect Dis Med Microbiol. 2020 Apr 30;2020:1042976. doi: 10.1155/2020/1042976. PMID: 32454916; PMCID: PMC7222550.
5. Nguyen TT, Nguyen DT, Vo TT, Dang OT, Nguyen BT, Pham DT, et al. Associations of obesity and dengue-associated mortality, acute liver failure and mechanical ventilation in children with dengue shock syndrome. Medicine (Baltimore). 2023 Nov 17;102(46):e36054. doi: 10.1097/MD.0000000000036054. PMID: 37986332; PMCID: PMC10659721.
6. Nguyen Tat T, Vo Hoang-Thien N, Nguyen Tat D, Nguyen PH, Ho LT, Doan DH, et al. Prognostic values of serum lactate-to-bicarbonate ratio and lactate for predicting 28-day in-hospital mortality in children with dengue shock syndrome. Medicine (Baltimore). 2024 Apr 26;103(17):e38000. doi: 10.1097/MD.0000000000038000. PMID: 38669370; PMCID: PMC11049702.
7. World Health Organization. (‎2009)‎. Dengue guidelines for diagnosis, treatment, prevention and control : new edition. World Health Organization. <https://iris.who.int/handle/10665/44188>

4. Previously identified risk factors (such as obesity and lactate levels) for adverse outcomes for DSS were not significant in your analysis. Do you have any explanation for this difference? (lines 359 to 362).

Lines 359-362: *“Notably, other covariates including obesity, underlying diseases, and serum lactate levels, which have been reported as important risk factors for adverse clinical outcomes in patients with DSS, were not significant in the LASSO and machine learning analysis.”*

**Response:** We are thankful for your comments. This is our answer for your queries.

These are possible explanations for the difference:

**For serum lactate:**

- First, high missing values of lactate data (41%) in our studied population, as shown in the first column in the following Pareto chart (**S1 Fig)**. Notably, ours is retrospective study and missing important data are inherent to this type of study design. We acknowledge that this is the weakness of our study.

- Second, most of our patients with DSS had clinically aggravated respiratory failure and required mechanical ventilation at the time points within 6-12 hours after PICU admission. Nevertheless, at this time, the lactate levels in most patients with severe DSS still did not change markedly. At 24h-48h after PICU admission, patients’ lactate levels would increase significantly. Nevertheless, we aimed to develop baseline model based on covariates collected from PICU admission and 6h after hospitalization. Hence, at this time almost blood lactate levels slightly changed .
- Upon our clinical supervision, serum lactate levels of severe dengue patients changed mostly significantly at the time point > 24h-48h after PICU admission. These data were supported from our published case series of patients with severe DSS on mechanical ventilation by Vo TL et al. [1].
- In this case series study of 67 DSS patients on MV, the dynamic changes in serum lactate levels from MV initiation (Time-0) and 24h (Time-24h) after MV are shown in the chart below [1].

(**Source:** Supplementary file, Vo TL et al, Pediatric Profound Dengue Shock Syndrome and Use of Point-of-Care Ultrasound (POCUS) During Mechanical Ventilation to Guide Treatment: Single-Center Retrospective Study, 2013-2021. Pediatr Crit Care Med. 2024). [1]

We can see that DSS patient’s lactate only changed markedly after MV commencement for > 24h. Nevertheless, from PICU admission to approximately 6h-12h (around MV commencement and setting), minimal change in blood lactate was seen compared to baseline value at PICU admission [1]. This means there was a lag time for blood lactate to show a significant increase. Nevertheless, we developed baseline (12h to 24h within PICU admission) prognostic model in this regard.

Therefore, a prospective study with dynamic modelling of serum lactate levels among DSS patients can be a good approach to elucidate the importance of serum lactate levels in DSS patients with respiratory failure requiring MV support.

**Considering the obesity variable, dengue severity and risk of MV:**

Several previous studies have shown that obesity is associated with the severity of dengue infection [2-5]. The possible pathogenesis is that obesity is associated with metabolism, the host immune response, overexpression of proinflammatory cytokines, and heightened vascular inflammation. These elements may compromise vascular endothelial cells, leading to increased capillary permeability and plasma leakage in obese dengue-infected patients [6-8].

Likewise, our research has recently reported an association between obesity and the risk of MV among patients with DSS using a conventional statistical method with multivariable logistic regression [5]. Notably, that study was restricted by the limited sample size, and nonlinearity of adjusted covariate analysis. Thus, the role of obesity in the risk of MV among DSS patients still cannot be determined clearly.

Significantly, a systematic review by Trang et al. in 2016 did not show an association between a patient’s nutritional status and obesity and dengue severity [9].

Therefore, the impact of nutritional status and obesity on dengue disease outcomes has remained unclear so far.

In this study, the advanced machine learning methods, particularly the support vector machine (SVM) model, can well manipulate the non-linearity of covariates. Therefore, the results of this study are more robust in determining that obesity was not associated with MV (in comparison with conventional statistical analysis).

**Reference**

1. Vo LT, Nguyen DT, Tran TN, Tran HH, Đoan TT, Pham TN, Mai TT, Nguyen QX, Nguyen TK, Nguyen TT, Burza S, Nguyen TT. Pediatric Profound Dengue Shock Syndrome and Use of Point-of-Care Ultrasound During Mechanical Ventilation to Guide Treatment: Single-Center Retrospective Study, 2013-2021. Pediatr Crit Care Med. 2024 Apr 1;25(4):e177-e185. Doi: 10.1097/PCC.0000000000003413. Epub 2023 Nov 15. PMID: 37966344; PMCID: PMC10986784.
2. Marón GM, Clará AW, Diddle JW, Pleités EB, Miller L, Macdonald G, Adderson EE. Association between nutritional status and severity of dengue infection in children in El Salvador. Am J Trop Med Hyg. 2010 Feb;82(2):324-9. Doi: 10.4269/ajtmh.2010.09-0365. PMID: 20134012; PMCID: PMC2813176.
3. Maneerattanasak S, Suwanbamrung C. Impact of Nutritional Status on the Severity of Dengue Infection Among Pediatric Patients in Southern Thailand. Pediatr Infect Dis J. 2020 Dec;39(12):e410-e416. Doi: 10.1097/INF.0000000000002839. PMID: 32773668; PMCID: PMC7654944.
4. Zulkipli MS, Rampal S, Bulgiba A, Peramalah D, Jamil N, See LLC, Zaki RA, Omar SFS, Dahlui M. Is there any association between body mass index and severity of dengue infection? Trans R Soc Trop Med Hyg. 2021 Jul 1;115(7):764-771. Doi: 10.1093/trstmh/trab021. PMID: 33587144.
5. Nguyen TT, Nguyen DT, Vo TT, Dang OT, Nguyen BT, Pham DT, et al. Associations of obesity and dengue-associated mortality, acute liver failure and mechanical ventilation in children with dengue shock syndrome. Medicine (Baltimore). 2023 Nov 17;102(46):e36054. Doi: 10.1097/MD.0000000000036054. PMID: 37986332; PMCID: PMC10659721.
6. Raqib R, Cravioto A. Nutrition, immunology, and genetics: future perspectives. Nutr Rev. 2009 Nov;67 Suppl 2:S227-36. Doi: 10.1111/j.1753-4887.2009.00244.x. PMID: 19906227.
7. Calabro P, Chang DW, Willerson JT, Yeh ET. Release of C-reactive protein in response to inflammatory cytokines by human adipocytes: linking obesity to vascular inflammation. J Am Coll Cardiol. 2005 Sep 20;46(6):1112-3. Doi: 10.1016/j.jacc.2005.06.017. PMID: 16168299.
8. Gallagher P, Chan KR, Rivino L, Yacoub S. The association of obesity and severe dengue: possible pathophysiological mechanisms. J Infect. 2020 Jul;81(1):10-16. Doi: 10.1016/j.jinf.2020.04.039. Epub 2020 May 12. PMID: 32413364.
9. Trang NTH, Long NP, Hue TTM, Hung LP, Trung TD, Dinh DN, Luan NT, Huy NT, Hirayama K. Association between nutritional status and dengue infection: a systematic review and meta-analysis. BMC Infect Dis. 2016 Apr 20;16:172. Doi: 10.1186/s12879-016-1498-y. PMID: 27097934; PMCID: PMC4839161.

5. Line 374 & 375 “Albumin solution should be administered to patients with severe DSS accompanied with unstable hemodynamic conditions” – Is this evidence-based?

**Response:** We thank for your significant point. This is our answer.

Low albumin levels have been reported as a prognostic factor for severe DSS, and low albumin levels indicate a high rate of plasma leakage [1,2]. Very low levels of blood albumin are also observed in children with DSS who have prolonged shock with multiple organ failure; these patients have a high mortality rate of nearly 70% despite intensive treatment [3,4]. Therefore, albumin supplementation not only increases intravascular volume, restores oncotic pressure, reduces the rate of fluid loss into the interstitial tissues, but also supports the restoration of the glycocalyx layer, which is known to be the main factor causing increased vascular permeability in DSS patients [5-8].

**References**

1.	World Health Organization. (‎2009)‎. Dengue guidelines for diagnosis, treatment, prevention and control : new edition. World Health Organization. <https://iris.who.int/handle/10665/44188>

2.	Sangkaew S, Ming D, Boonyasiri A, Honeyford K, Kalayanarooj S, Yacoub S, Dorigatti I, Holmes A. Risk predictors of progression to severe disease during the febrile phase of dengue: a systematic review and meta-analysis. Lancet Infect Dis. 2021 Jul;21(7):1014-1026. doi: 10.1016/S1473-3099(20)30601-0. Epub 2021 Feb 25. PMID: 33640077; PMCID: PMC8240557.

3.	Vo, Luan Thanh MD; Do, Viet Chau MD; Trinh, Tung Huu MD; Vu, Thien PhD; Nguyen, Thanh Tat MD. Combined Therapeutic Plasma Exchange and Continuous Renal Replacement Therapy in Children With Dengue-Associated Acute Liver Failure and Shock Syndrome: Single-Center Cohort From Vietnam*. Pediatric Critical Care Medicine 24(10):p 818-828, October 2023. | DOI: 10.1097/PCC.0000000000003304

4.	Kye Mon K, Nontprasert A, Kittitrakul C, Tangkijvanich P, Leowattana W, Poovorawan K. Incidence and Clinical Outcome of Acute Liver Failure Caused by Dengue in a Hospital for Tropical Diseases, Thailand. Am J Trop Med Hyg. 2016 Dec 7;95(6):1338-1344. doi: 10.4269/ajtmh.16-0374. Epub 2016 Oct 17. PMID: 27928082; PMCID: PMC5154447.

5.	Saravi B, Goebel U, Hassenzahl LO, Jung C, David S, Feldheiser A, Stopfkuchen-Evans M, Wollborn J. Capillary leak and endothelial permeability in critically ill patients: a current overview. Intensive Care Med Exp. 2023 Dec 20;11(1):96. doi: 10.1186/s40635-023-00582-8. PMID: 38117435; PMCID: PMC10733291.

6.	Durbin AP. Dengue vascular leak syndrome: insights into potentially new treatment modalities. J Clin Invest. 2019 Oct 1;129(10):4072-4073. doi: 10.1172/JCI131170. PMID: 31449055; PMCID: PMC6763244.

7.	Malavige GN, Ogg GS. Pathogenesis of vascular leak in dengue virus infection. Immunology. 2017 Jul;151(3):261-269. doi: 10.1111/imm.12748. Epub 2017 May 24. PMID: 28437586; PMCID: PMC5461104.

8.	Aldecoa C, Llau JV, Nuvials X, Artigas A. Role of albumin in the preservation of endothelial glycocalyx integrity and the microcirculation: a review. Ann Intensive Care. 2020 Jun 22;10(1):85. doi: 10.1186/s13613-020-00697-1. PMID: 32572647; PMCID: PMC7310051.

6. Do you have any explanation for why increased hematocrit is a protective factor for MV?

**Response:** We thank for your comment. We will clarify this point.

Based on our recent publication on the prognostic modelling of MV in patients with DSS, we have shown that elevated hematocrit is a protective factor of MV [3].

As presented in Table 4, from the published article by Nguyen TT et al., “*Associations of obesity and dengue-associated mortality, acute liver failure and mechanical ventilation in children with dengue shock syndrome*” in Medicine (Baltimore) in 2023 [3], and it can be seen from **Table 4 (Row 5)** from the multivariable logistic analysis, HCT yielded an adjusted odds ratio, OR = 0.91 (0.87-0.95), P < 0.001. This can be interpreted that each 1% increase in HCT reduces the risk for MV requirement by 9%. This also means that HCT is protective against mechanical ventilation.

**Reference**

[3] Nguyen TT, Nguyen DT, Vo TT, Dang OT, Nguyen BT, Pham DT, Nguyen TT, Duong YN, Doan DH, Nguyen TH, Ho LT, Nguyen PH, Phan DN, Tran TV, Nguyen TK, Luong DC, Pham AT, Dinh TT, Do VC, Vo LT. Associations of obesity and dengue-associated mortality, acute liver failure and mechanical ventilation in children with dengue shock syndrome. Medicine (Baltimore). 2023 Nov 17;102(46):e36054. Doi: 10.1097/MD.0000000000036054. PMID: 37986332; PMCID: PMC10659721.

Further evidence to support the statement “**HCT is a protective factor against MV**”, stemming from our SHAP analysis for HCT covariable, as presented in the following SHAP plot:

It can be seen from this SHAP plot, in general, increased HCT levels corresponds to decreased SHAP values. A cut-off HCT > 48% corresponds to lower SHAP values, indicating a lower risk of MV, despite a slight increase in SHAP when HCT was > 57%.

Therefore, these data support that increased HCT is a protective factor against MV in patients with DSS.

We have also added a citation [3] from the appropriately published data to support this point.

**Lines 393-394,** in the revision manuscript**.**

**Reference**

[3] Nguyen TT, Nguyen DT, Vo TT, Dang OT, Nguyen BT, Pham DT, et al. Associations of obesity and dengue-associated mortality, acute liver failure and mechanical ventilation in children with dengue shock syndrome. Medicine (Baltimore). 2023; 102 (46): e36054. Doi: 10.1097/MD.0000000000036054. PMID: 37986332; PMCID: PMC10659721

7. Validation of this score with prospective studies is necessary before recommending this score in clinical practice (line 408).

**Response:** We are thankful for your comment. We totally agree that “validation of this score with prospective studies is necessary before recommending this score in clinical practice”

We have added your suggested point in the revision manuscript (**Lines 409-410).**

We have answered all the questions by all the reviewers and have offered corrections as needed. We are thankful for your significant comments that have made our study more comprehensive to the readers.

We look forward to your feedback and answer any further questions.

Many thanks,

Best regards,

Dr Thanh Nguyen Tat
